# Characterization of the diethyl phthalate-degrading bacterium *Sphingobium yanoikuyae* SHJ

**DOI:** 10.1016/j.dib.2018.09.033

**Published:** 2018-09-18

**Authors:** Yan Wang, Hui Liu, Yue’e Peng, Lei Tong, Liang Feng, Kesen Ma

**Affiliations:** a State Key Laboratory of Biogeology and Environmental Geology, China University of Geosciences, Wuhan 430074, China; b Department of Environmental Sciences and Engineering, School of Environmental Studies, China University of Geosciences, Wuhan 430074, China; c School of Materials Science and Chemistry, China University of Geosciences, Wuhan 430074, China; d Department of Biology, University of Waterloo, Waterloo, Ontario, Canada N2L 3G1

**Keywords:** Sphingobium yanoikuyae, Gene sequence, Genome, Diethyl phthalate, Biodegradation

## Abstract

A newly isolated bacterial strain SHJ was found to be capable of degrading diethyl phthalate (DEP) very efficiently. Its growth characteristics and 16S rDNA gene sequence were analyzed. Its whole genome was also sequenced. Strain SHJ was identified as *Sphingobium yanoikuyae* SHJ.

**Specifications table**

**Table:** 

**Subject area**	Biology
**More specific subject area**	Microbial characterization, identification and phylogenetic analysis
**Type of data**	Table, figure
**How data was acquired**	Microscope, SEM, DNA sequencing, bioinformatics
**Data format**	Raw, analyzed and deposited
**Experimental factors**	Strain SHJ was cultured for observation and 16S rDNA gene sequencing analysis
**Experimental features**	A new microbe was isolated, cultured, observed under a scanning electron microscope. The morphology of its colonies on agar plate was described. Its 16S rDNA gene was sequenced, for which phylogenetic analysis was performed.
**Data source location**	Sample was collected at 30°28′19″N, 113°59′13″E (longitude, latitude), Wuhan, Hubei, China
**Data accessibility**	With this article, GenBank accession number JFFT01000000, DDBJ/EMBL/GenBank under the accession JFFT00000000

**Value of the data**

• The whole genome sequence data of strain SHJ is available by its accession number.
• Characterization and identification of the newly isolated Sphingobium yanoikuyae SHJ.
• Biodiversity with capability of bio-remediating phthalate esters-contaminated aquifer.

## 1 Data

**Fig. 1:**
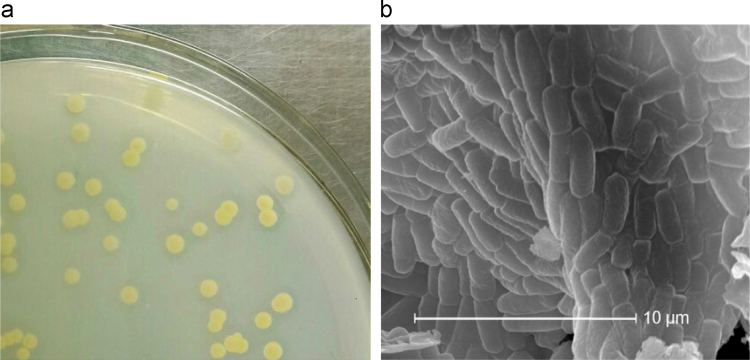
The growth of strain SHJ on NB agar plate containing 400 mg L^−1^ DEP (a) and its cell morphology under a scanning electron microscope (b).

**Fig. 2:**
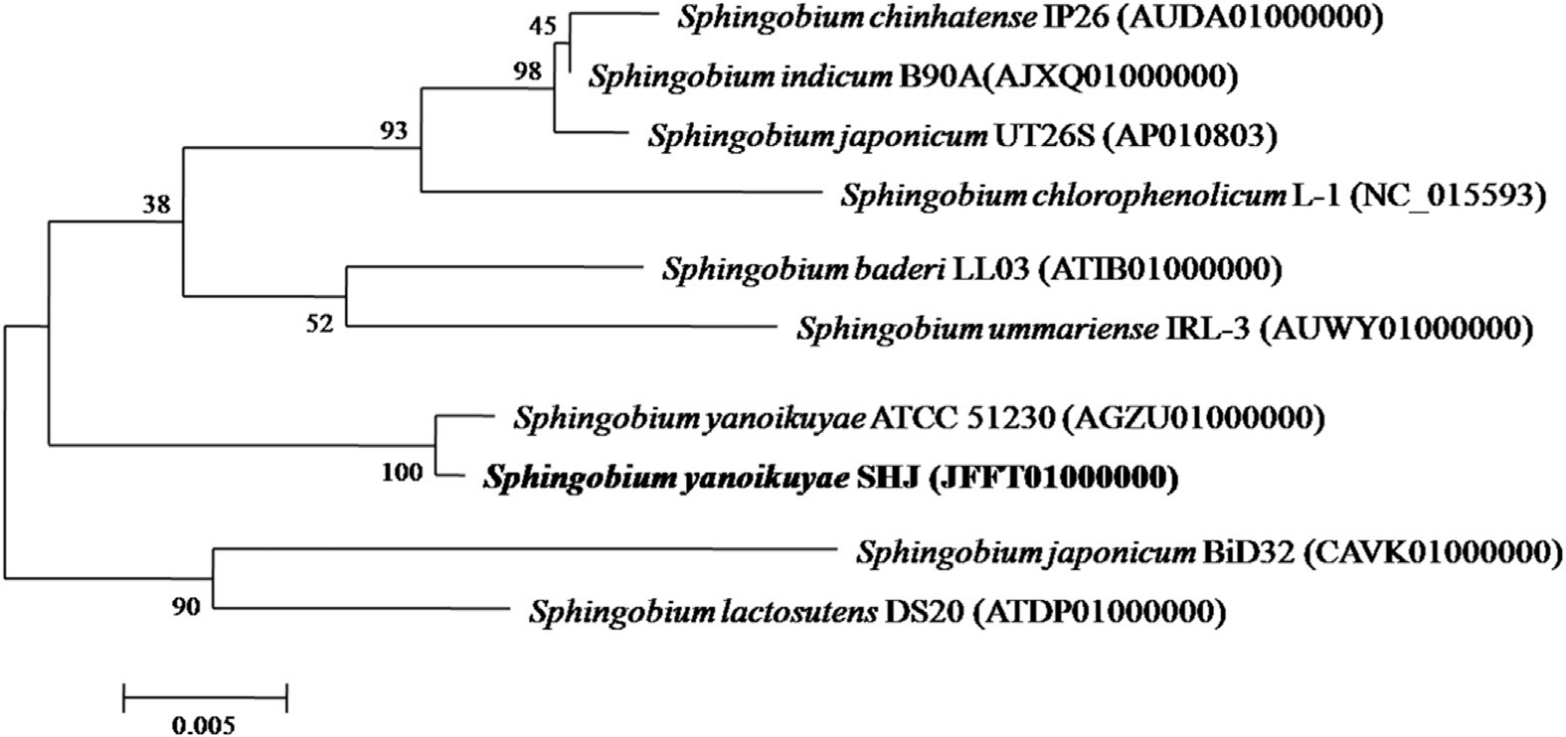
Phylogenetic analysis of 16S rDNA sequences. The tree was built using the maximum-likelihood method with the Hasegawa-Kishino-Yano model assuming non-uniformity of evolutionary rates among sites (https://www.megasoftware.net/index.php). Bootstrap analysis with 1000 replicates was performed to assess the support of the clusters. The corresponding GenBank accession numbers are displayed in parentheses.

A new bacterium strain SHJ was isolated from the shallow aquifer sediment of Jianghan plain, Hubei, China. It grew on NB agar plate containing 400 mg L^−1^ DEP as sole carbon source and appeared to be yellow colony (Fig. 1a), and it was observed to be short rod under a scanning electron microscope (Fig. 1b). It was found to be capable of degrading DEP very efficiently under simulated shallow aquifer (SSA) conditions which are dark, oxygen-limited, at pH 7 and 18 °C [1]. However, the most well-known DEP-degrading bacterial isolates that are purely aerobic are listed in Table 1. Classification and general features of the strain SHJ were listed in Table 2. Its 16S rDNA gene sequence (GenBank accession number JFFT01000000) showed the highest similarity with *Sphingobium yanoikuyae* ATCC 51230 (Fig. 2). Therefore, strain SHJ was classified as Sphingobium yanoikuyae SHJ. The Whole Genome Shotgun project of S. yanoikuyae SHJ has been deposited at DDBJ/EMBL/GenBank under the accession JFFT00000000 and the release date of its GenBank Data is February 28, 2017.

**Table 1:** Several DEP-degrading bacterial strains isolated from various environments.

**Species**	**Isolation**	**DEP (mg L**^**−1**^**)**	**Performance**	**References**
*Bacillus subtilis* 3C3	Soil	100	60% after 24 h	Navacharoen et al. [2]
*Bacillus thuringiensis*	Agricultural soil	400	88% after 80 h	Surhio et al. [3]
*Rhodococcus* sp. L4	Activated sludge	100	100% after 6 days	Lu et al. [4]
*Mycobacterium* sp YC-RL4	Petroleum-contaminated soil	50	100% after 5 days	Ren et al. [5]
*Acinetobacter* sp. LMB-5	Vegetable greenhouse soil	100	95% after 45 h	Fang et al. [6]
*Acinetobacter* sp. JDC-16	River sludge	500	100% after 27 h	Liang et al. [7]
*Pseudomonas fluoresences* FS1	Activated sludge at a petrochemical factory	100	100% after 36 h	Zeng et al. [8]
*Pleurotus ostreatus*	Forest soil	100	100% after 8 days	Hwang et al. [9]
*Gordonia alkanivorans* YC-RL2	Petroleum-contaminated soil	100	100% after 7 days	Nahurira et al. [10]
*Sphingomonas* sp. C28242	Activated sludge	450	100% after 120 h	Fang et al. [6]
*Sphigomonas* sp. DK4	River sediment	100	56% after 7 days	Chang et al. [11]
*Corynebacterium* sp.O18	Petrochemical sludge	100	100% after 7 days	Chang et al. [11]

**Table 2:** Classification and general features of *Sphingobium yanoikuyae* SHJ according to the MIGS (miRNA-induced gene silencing) recommendation.

**MIGS ID**	**Property**	**Term**	**Evidence code**^a^
	Current classification	Domain *Bacteria*	TAS [12]
		Phylum *Proteobacteria*	TAS [13]
		Class *Alphaproteobacteria*	TAS [14]
		Order *Sphingomonadales*	TAS [15]
		Family *Sphingomonadaceae*	TAS [16]
		Genus *Sphingobium*	TAS [17]
		Species *yanoikuyae*	TAS [17]
	Gram stain	Gram-negative	IDA
	Cell shape	Short rod-shaped	IDA
	Motility	Non-motile	IDA
	Sporulation	Non-spore-forming	IDA
	Temperature range	13–30 °C	IDA
	Optimum temperature	28 °C	IDA
	pH range; Optimum	6–9;6.8	IDA
	Carbon source	L-arabinose, D-xylose, galactose, Salicin, mannose, D-turanose, and caprate	TAS [17]
	Energy source	Chemoheterotrophic	TAS [17]
MIGS-6	Habitat	Sediments	IDA
MIGS-6.3	Salinity	Slight Halophilic	IDA
MIGS-22	Oxygen	Facultative aerobe	IDA
MIGS-15	Biotic relationship	Free living	IDA
MIGS-14	Pathogenicity	None	NAS
MIGS-4	Geographic location	Caidian District, Wuhan, Hubei, China	IDA
MIGS-5	Sample collection time	2008	IDA
MIGS-4.1	Latitude	30°28′19″ N	NAS
MIGS-4.2	Longitude	113°59′13″ E	NAS
MIGS-4.3	Depth	2.2 m	NAS
MIGS-4.4	Altitude	24 m	NAS

a Evidence codes - IDA: Inferred from Direct Assay; TAS: Traceable Author Statement (i.e., a direct report exists in the literature); NAS: Non-traceable Author Statement (i.e., not directly observed for the living, isolated sample, but based on a generally accepted property for the species, or anecdotal evidence). These evidence codes are from the Gene Ontology project.

## 2 Experimental design, materials and methods

### 2.1 Chemicals and reagents

DEP was purchased from Tianjin Hengxing Chemical Reagent Co., Ltd., China. DEP standard solutions were prepared at various concentrations in methanol and kept in dark at 4 °C.

### 2.2 DEP-degrading bacterial strain

The DEP-degrading strain SHJ was isolated from the sediments collected from the quaternary shallow aquifer from a depth of 2.2 m in Jianghan Plain, Hubei, China, with a precise GPS location of 30°28′19″N, 113°59′13″E (longitude, latitude). The strain SHJ was grown using the method described previously [18]. It was pre-grown for 24 h at pH 7.2 and 30 °C in nutrient broth (NB), which contained peptone 5 g L^−1^, beef extract 3 g L^−1^, NaCl 5 g L^−1^. Nutrient agar plates were prepared using NB supplemented with agar (1.5%). NB-DEP agar plate was prepared by diffusing 400 mg L^−1^ DEP solution into the nutrient agar medium. All media were sterilized for 20 min at 121 °C before inoculation. Detection and identification of DEP degradation intermediates ethyl methyl phthalate (EMP), monoethyl phthalate (MEP), monomethyl phthalate (MMP) and phthalic acid (PA) was carried out as described previously [1].

### 2.3 Identification of strain SHJ

Colonies of the strain SHJ on NB agar plate were picked for Gram staining, and the morphology of the strain was observed using an optical microscope.

Microbial identification and phylogenetic analysis of strain SHJ were performed by 16S rDNA gene sequencing. One ml overnight culture of bacterium grown in NB media in a rotary shaker (150 rpm) at 30 °C was centrifuged at 6000×*g* for 10 min. The cells obtained were washed three times using sterile water and re-suspended in sterile water. Genome DNA was extracted from the isolate using UltraClean^®^ Microbial DNA Isolation Kit (MoBio, USA) according to the manufacturer׳s protocol. 16S rDNA gene of the strain SHJ was amplified from its genomic DNA by using PCR procedures [18]. The bacterial universal primers F27 and R1492 were used for amplifying the full length of 16S rRNA gene fragments. The Shanghai Personal Biotechnology Co., Ltd performed the sequencing and assembly of strain SHJ using Illumina MiSeq sequencing platform, and gene prediction and annotation were completed using National Center for Biotechnology Information (NCBI) Prokaryotic Genome Annotation Pipeline (PGAP, https://www.ncbi.nlm.nih.gov/genome/annotation_prok/) [1]. The 16S rDNA gene sequence of strain SHJ was searched against GenBank database under the accession JFFT00000000 using BLASTn at the website of NCBI (http://www.ncbi.nlm.nih.gov/BLAST/). Based on the 16S rDNA gene sequences obtained, phylogenetic analysis of strain SHJ was performed by molecular evolutionary genetics analysis (MEGA 6, https://www.megasoftware.net/index.php) after all sequences alignment by using Clustal W (https://www.ebi.ac.uk/Tools/msa/clustalw2/).
