# Supplementary material for: Characterization of the diethyl phthalate-degrading bacterium Sphingobium yanoikuyae SHJ
Source: Data Brief. 2018 Sep 18;20:1758–63. doi: 10.1016/j.dib.2018.09.033 (PMC6161454; doi:10.1016/j.dib.2018.09.033)
Supplement: Supplementary file 1 — Supplementary material. [file mmc1.docx]

**Conflict of Interest From**

The authors have no conflict of interest.
